# Technical Report: Biodegradable Spacer Device for Irreparable Rotator Cuff Tears

**DOI:** 10.7759/cureus.3307

**Published:** 2018-09-14

**Authors:** MN Baig, Orna A Glynn, Kenneth Kaar

**Affiliations:** 1 Trauma & Orthopaedic Surgery, Galway University Hospital, Galway, IRL; 2 Trauma & Orthopaedics, Galway University Hospital, Galway, IRL

**Keywords:** spacer, rotator cuff

## Abstract

The practice of medicine involves gauging the efficacy of current treatments, the associated complications, and the scope for improvement. Rotator cuff tears are a common problem, and, over the years, a variety of treatments have been used to help the patients. We describe a technique using a biodegradable balloon spacer device for treating massive irreparable rotator cuff tears.

## Introduction

“Rotator cuff” is a term used for a group of four muscles in the shoulder girdle originating from the scapula and inserting into the greater tuberosity. Rotator cuff muscles act upon the glenohumeral joint, and they stabilize and control the movements of the shoulder. Rotator cuff tears are one of the more common causes of shoulder pain and disability. The tears can be of partial thickness or full thickness and can be classified as small, medium, large, or massive, according to their size. The prevalence of full-thickness tears is 28% in patients over 60 years old and 65% in patients over 70 years old [1].

The most common muscle torn in rotator cuff injuries is the supraspinatus. Treatment options usually include physiotherapy, analgesia, and an open or arthroscopic repair of the rotator cuff.

## Technical report

**Figure 1:**
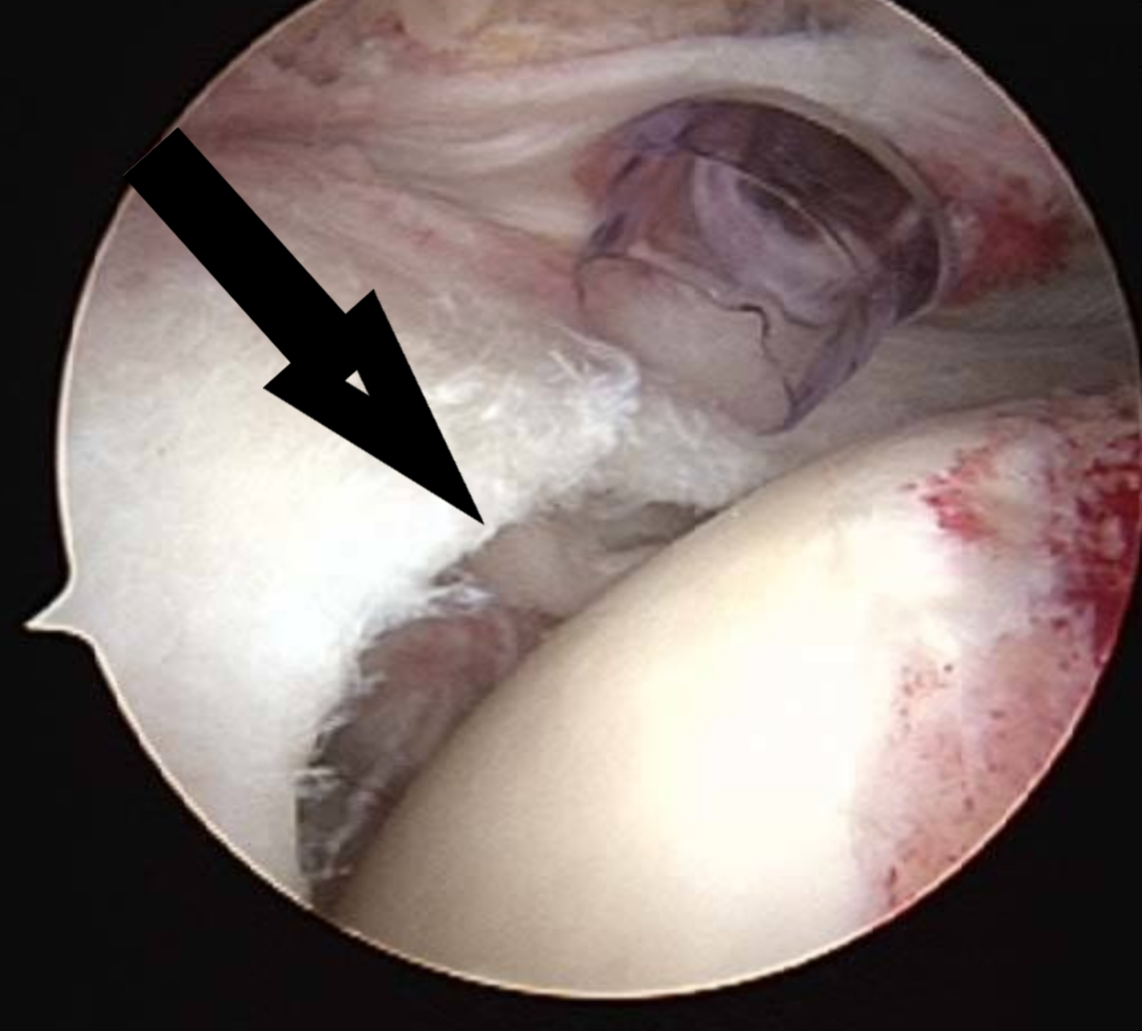
Rotator cuff tear

**Figure 2:**
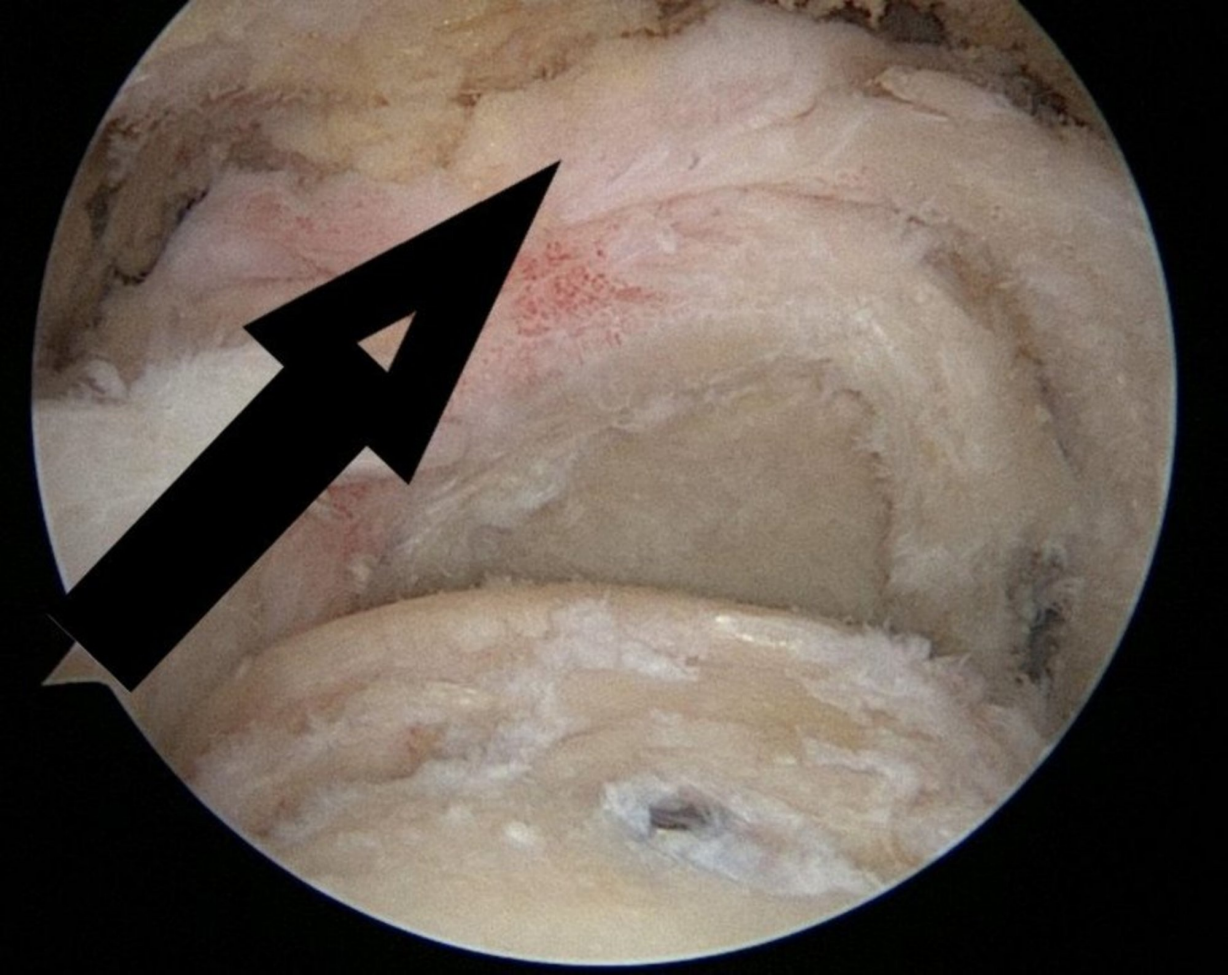
Massive rotator cuff tear with retraction

In some cases, the rotator cuff tear is complete (full thickness) with massive retraction, and it is not possible to repair it with conventional methods (Figures 1-2). In these circumstances, a new device—a biodegradable balloon—can be used as a spacer inserted between the acromion and the humeral head [2].

**Figure 3:**
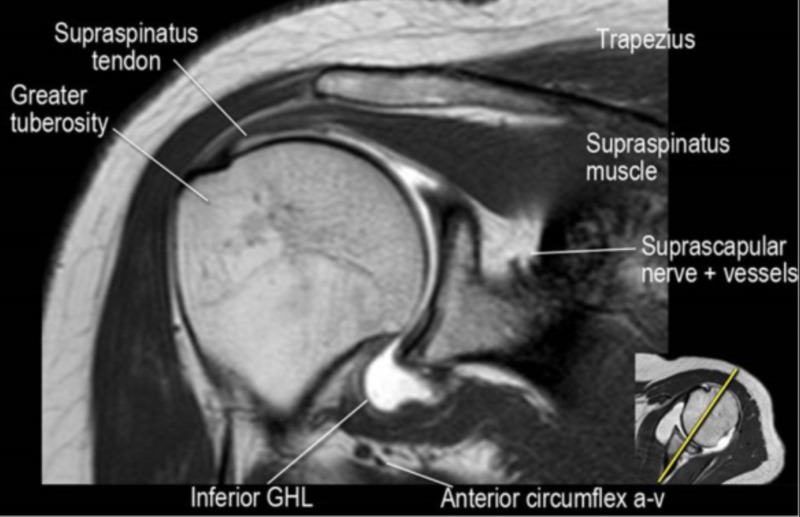
Normal shoulder MRI Magnetic resonance imaging (MRI) showing normal anatomy. GHL: glenohumeral ligament; a-v: artery/vein

**Figure 4:**
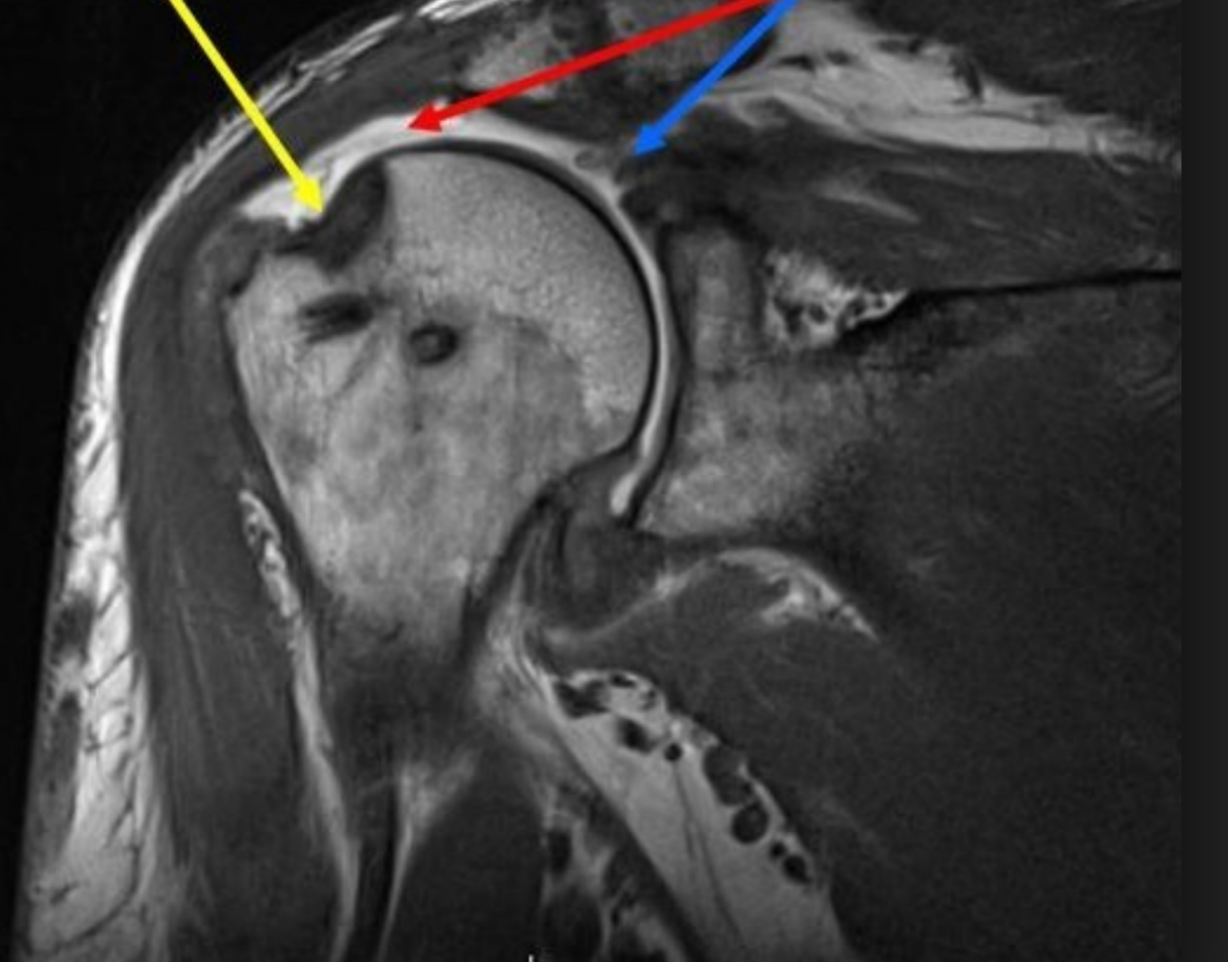
Rotator cuff tear - MRI Magnetic resonance imaging (MRI) showing a rotator cuff tear. Yellow - insertion site of supraspinatus; Red - space where the supraspinatus is present; Blue - retracted tendon of the supraspinatus muscle.

The rotator cuff tear can be partial or complete (the most common being a supraspinatus tear). In the following images, there is a normal magnetic resonance imaging (MRI) shoulder and an MRI showing a supraspinatus tear (Figures 3-4).

**Figure 5:**
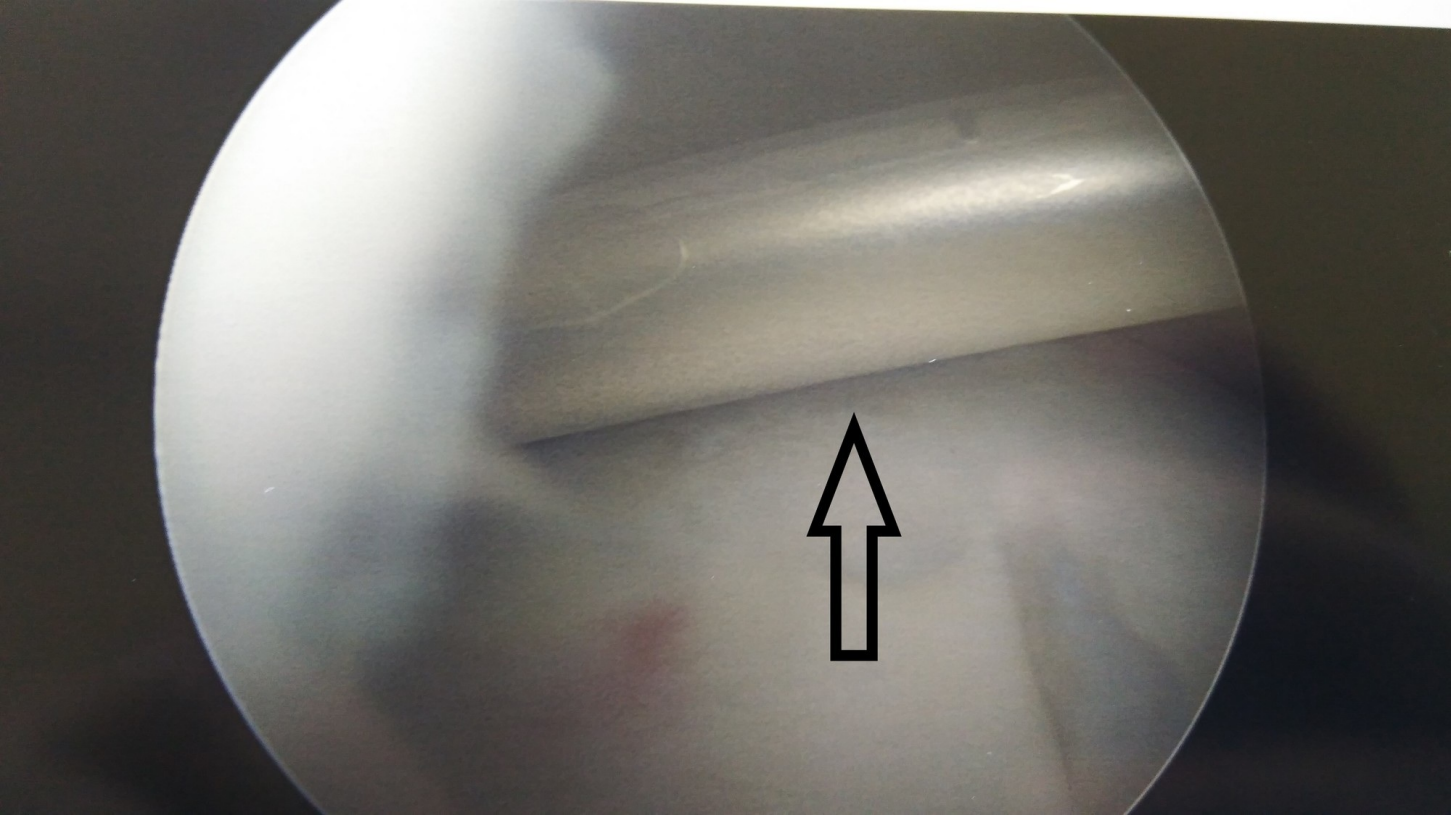
Spacer balloon Introduction in-vivo, unfolded. InSpace device (InSpace; OrthoSpace, Kfar Saba, Israel).

The spacer balloon device is used for irreparable massive rotator cuff tears. The device we use is the Inspace biodegradable balloon (OrthoSpace, Kfar Saba, Israel), which consists of an introducer with a balloon mounted on top. It is connected with a 60 ml syringe via connector tubing and introduced into the shoulder through an arthroscopic portal opening in the subacromial space between the humeral head and the acromion. Once positioned, the spacer (balloon) is inflated with saline to allow smooth and frictionless gliding. To use the device, the arthroscope is introduced into the subacromial space through a posterior incision. The subacromial space is then debrided and decompressed via an arthroscopic shaver. Next, a standard lateral incision is made through which a specially marked device is passed to measure the size of the subacromial space. The appropriate spacer is selected based on the determined size. An introducer device with the spacer mounter is introduced through the lateral incision (Figure 5).

**Figure 6:**
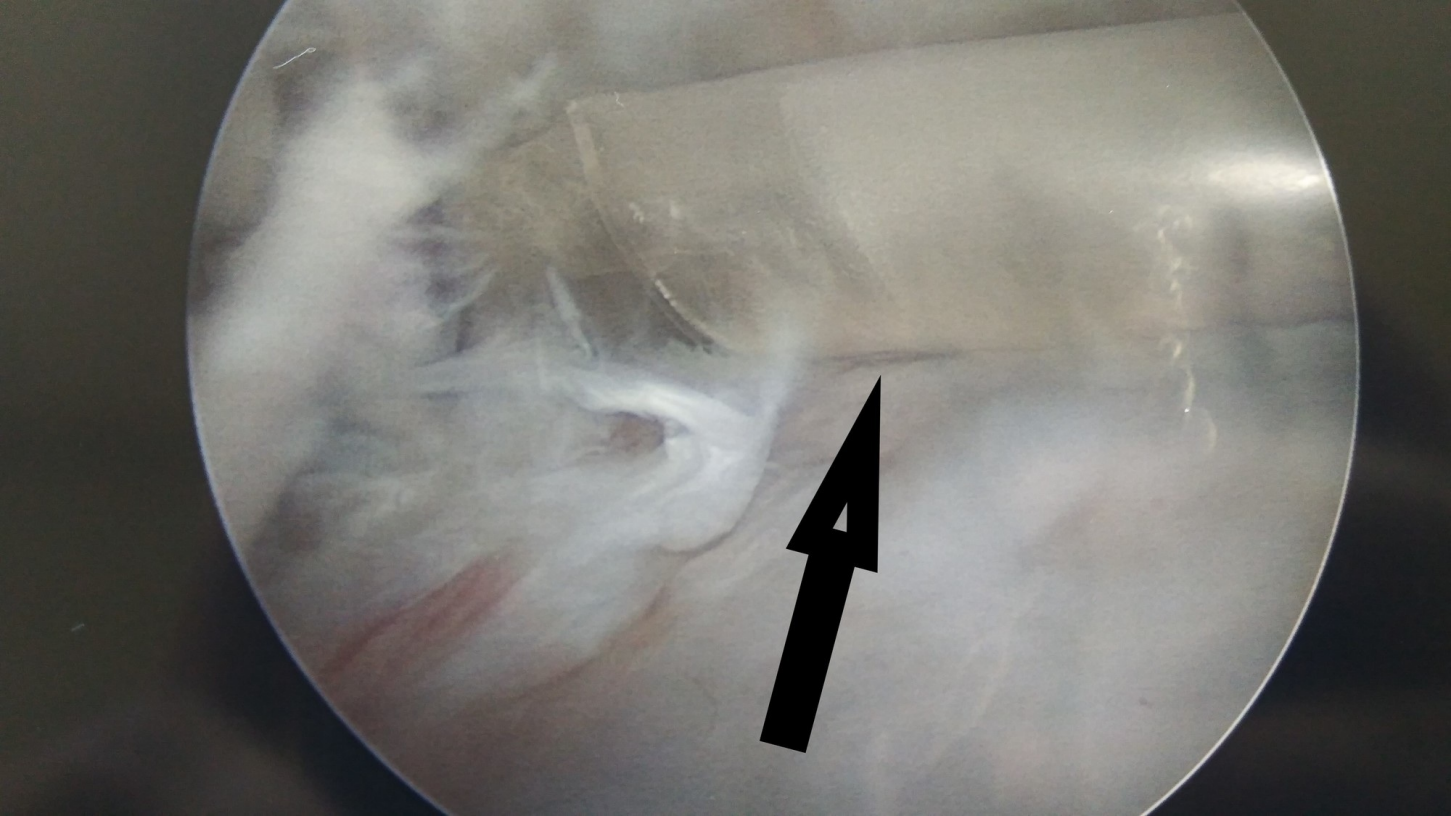
Unwrapping of spacer balloon The balloon device injected with saline. InSpace device (InSpace; OrthoSpace, Kfar Saba, Israel).

**Figure 7:**
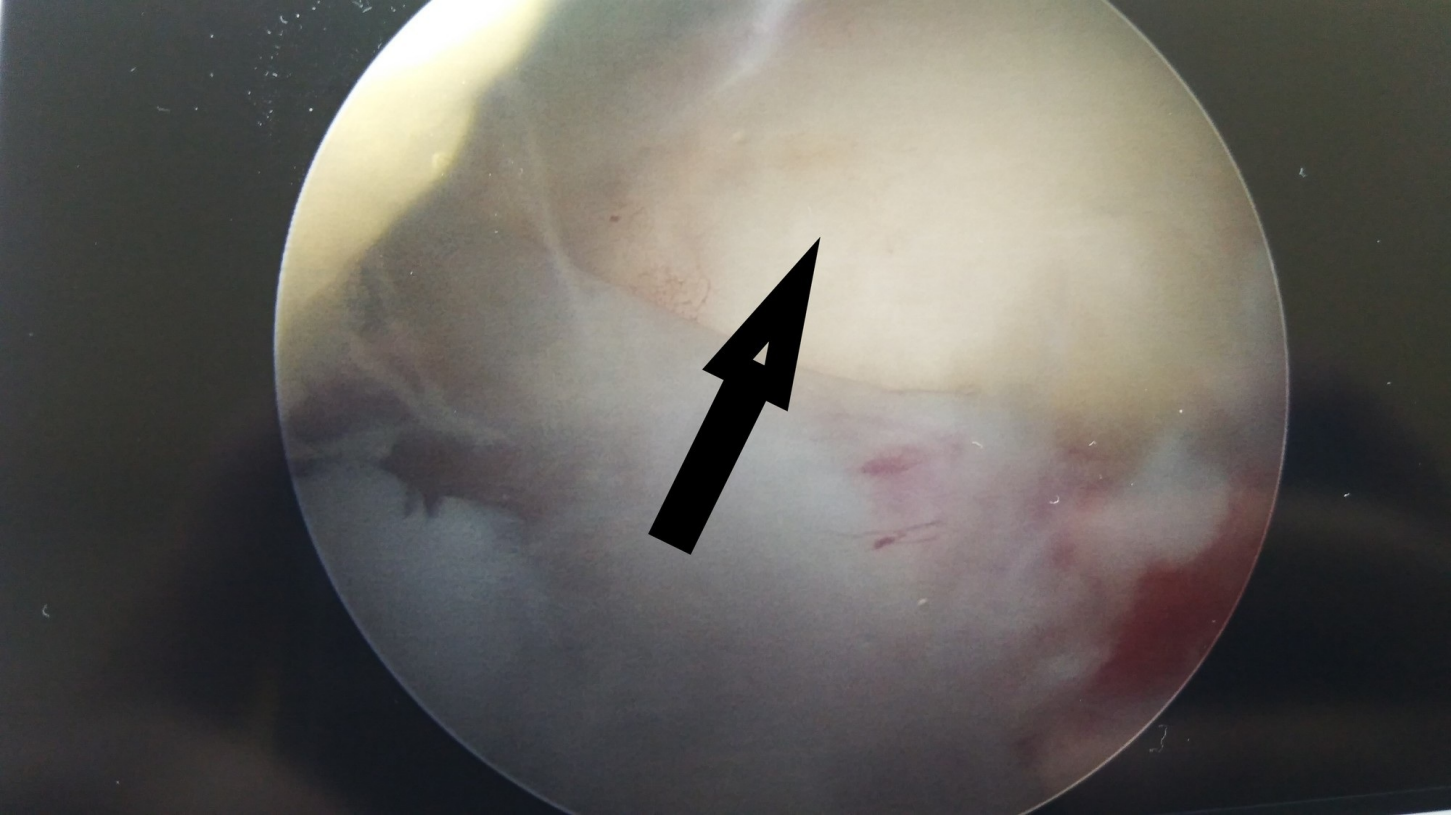
Full expansion of spacer Fully expanded balloon spacer in-vivo. InSpace device (InSpace; OrthoSpace, Kfar Saba, Israel).

Once satisfied with the positioning of the device, the surgeon fills the device with fluid through the connector tubing. The balloon unfolds gradually and inflates (Figures 6-7). As expected, different sizes of the device require different amounts of saline to inflate.

**Figure 8:**
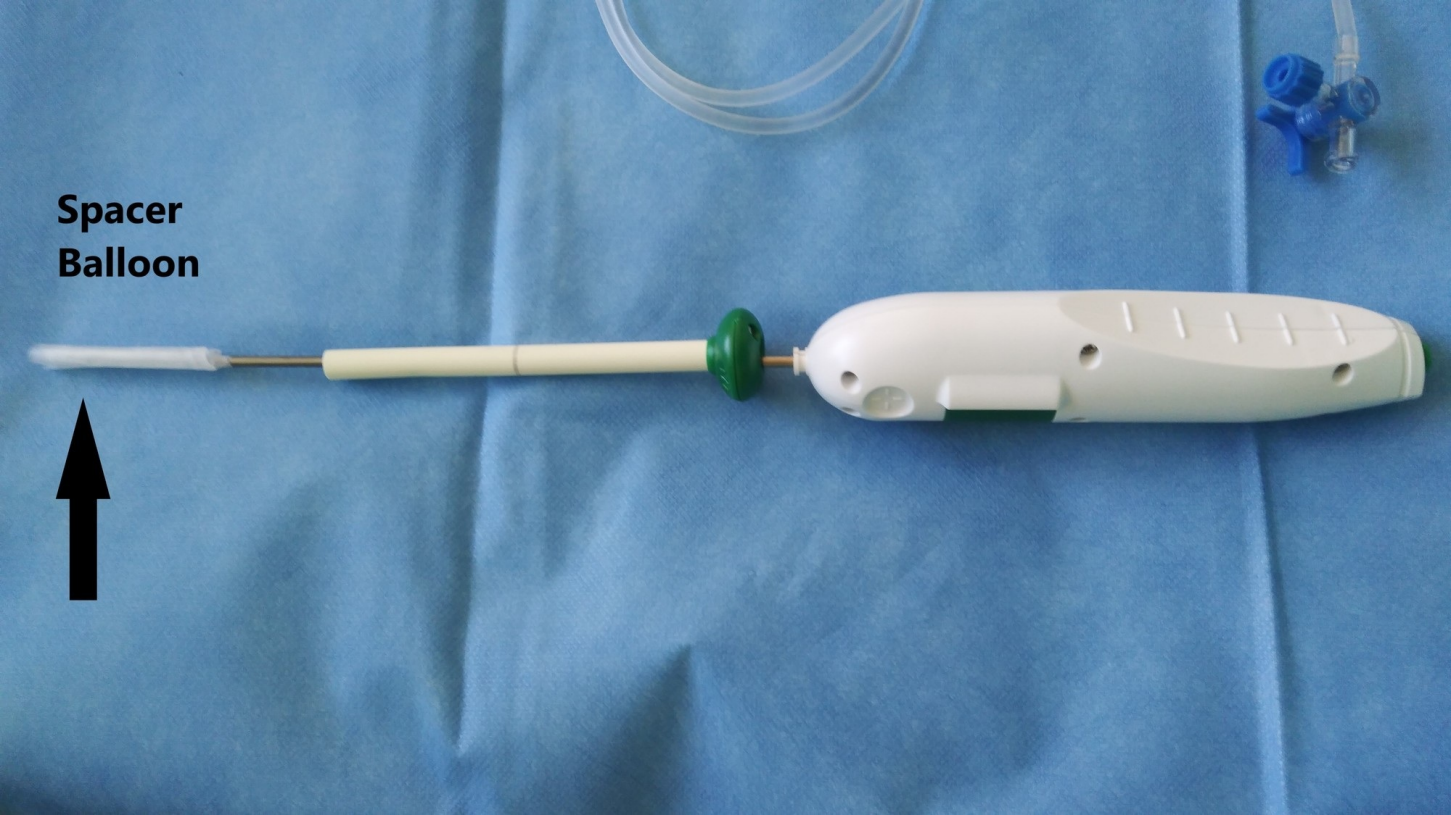
Spacer balloon device Spacer balloon device in-vitro. InSpace device (InSpace; OrthoSpace, Kfar Saba, Israel).

**Figure 9:**
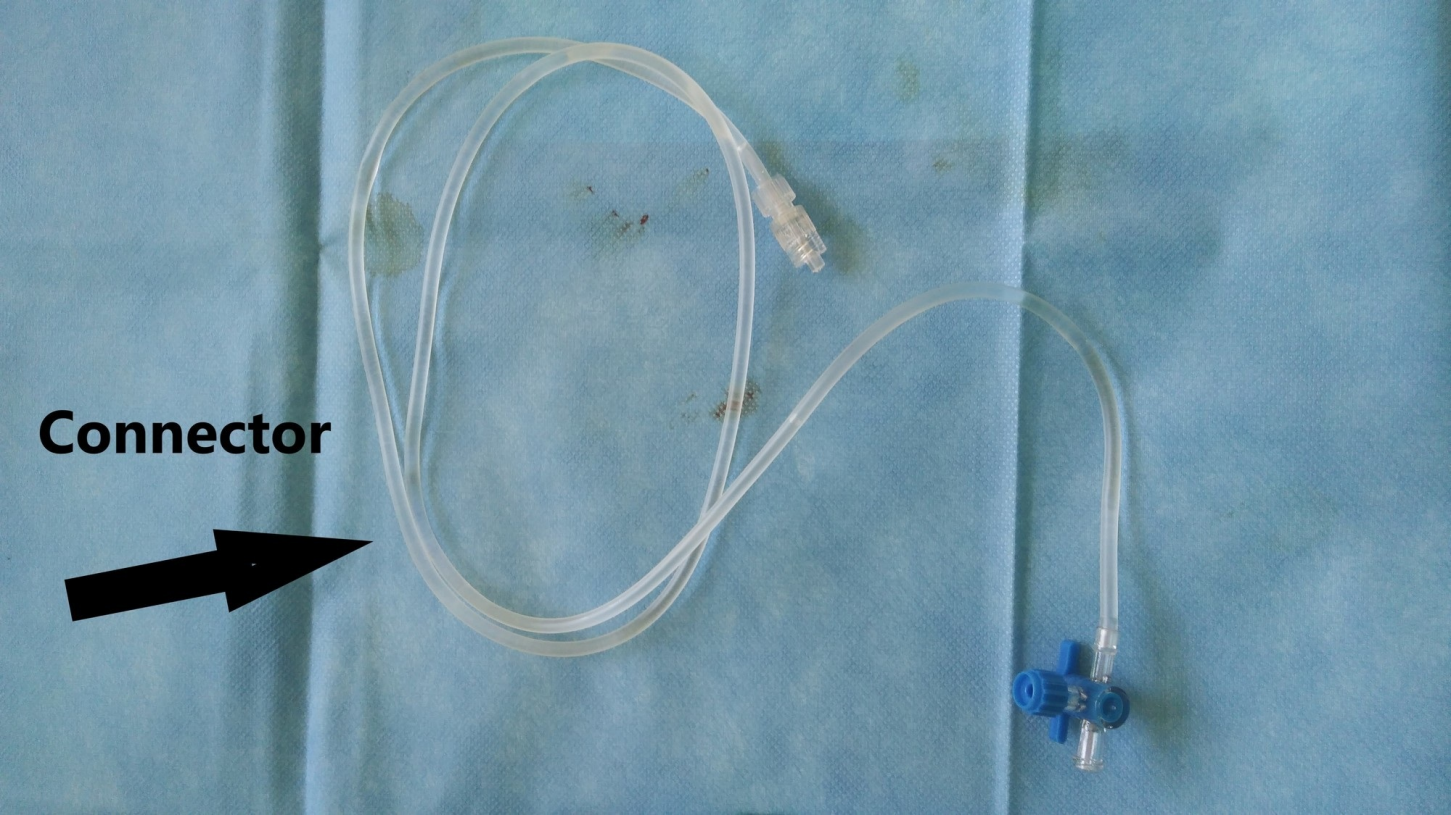
Connector In-vitro connector. InSpace device (InSpace; OrthoSpace, Kfar Saba, Israel).

**Figure 10:**
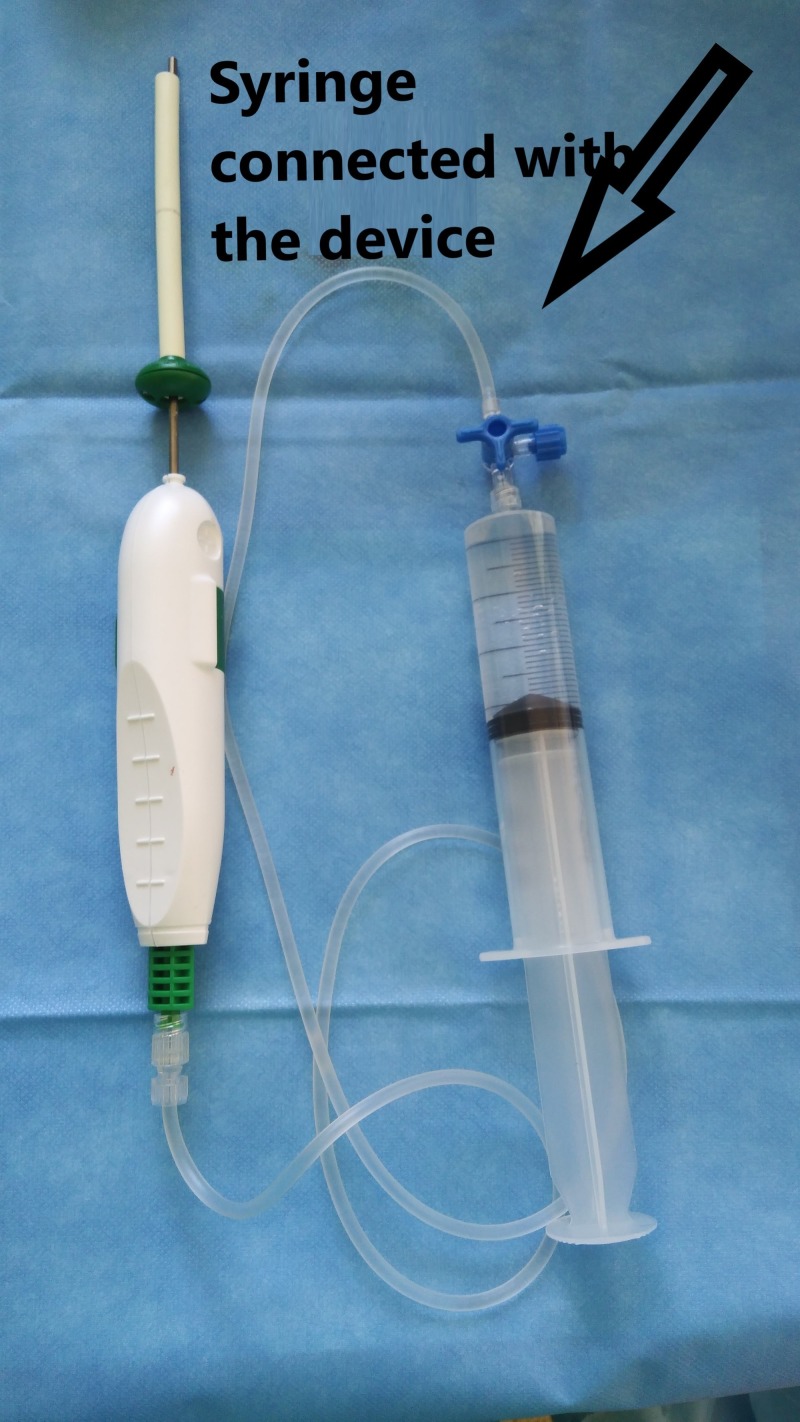
Spacer device connected to syringe In-vitro device consisting of a balloon spacer and a syringe with saline. InSpace device (InSpace; OrthoSpace, Kfar Saba, Israel).

**Figure 11:**
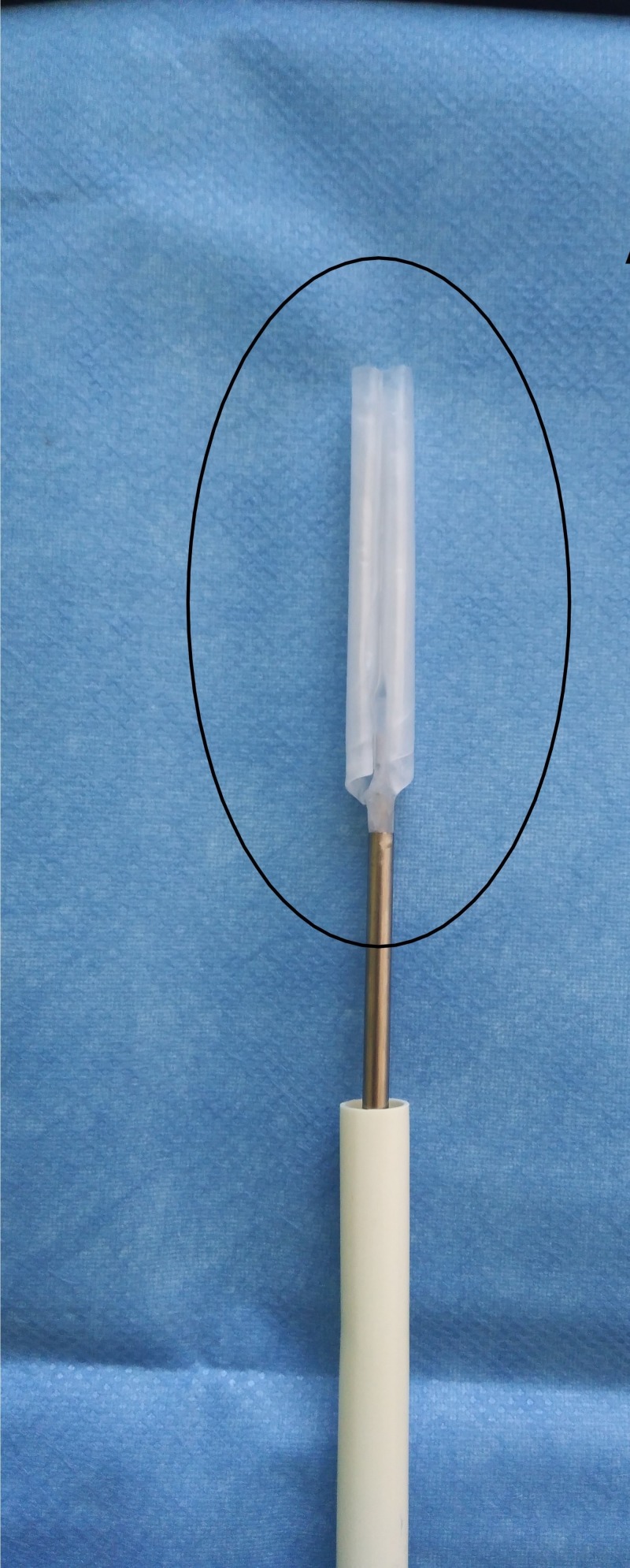
Spacer balloon Unfolded spacer balloon in-vitro. InSpace device (InSpace; OrthoSpace, Kfar Saba, Israel).

**Figure 12:**
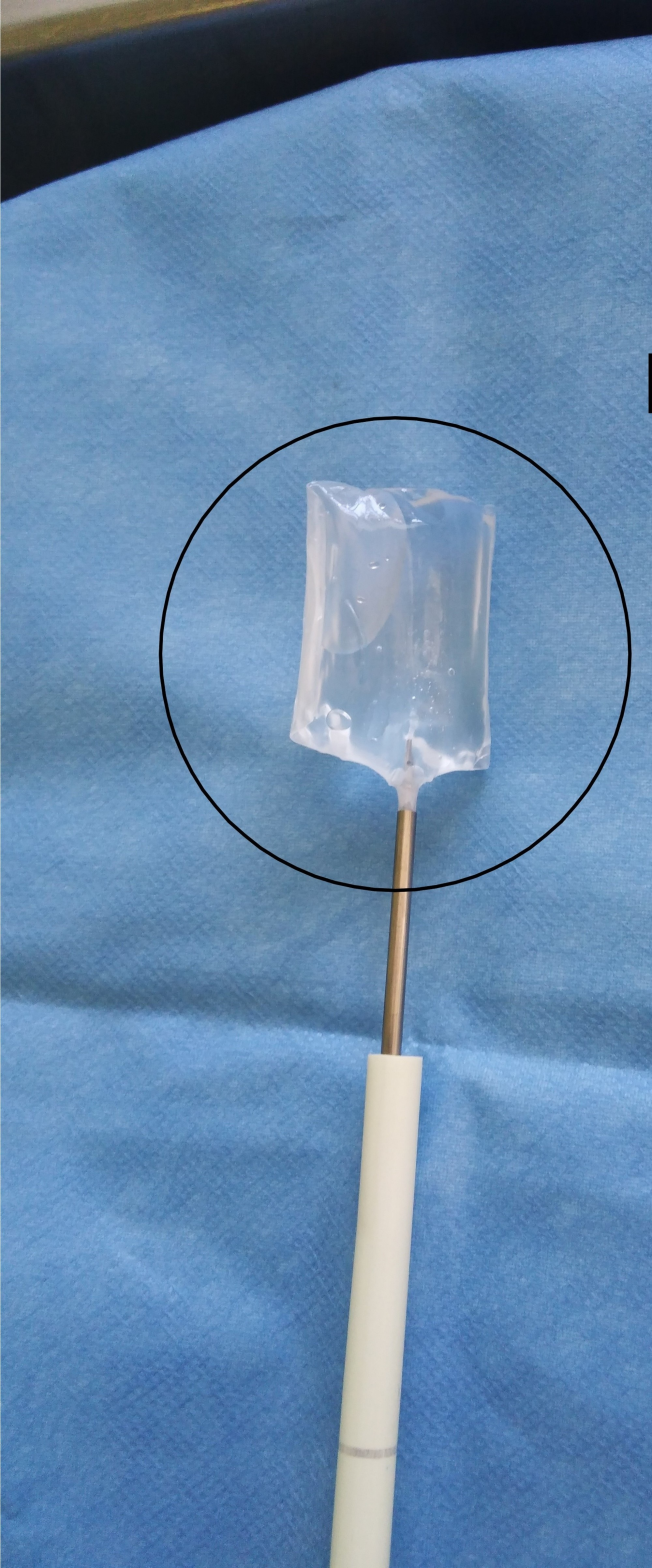
Spacer balloon inflation Continuation of spacer - in-vitro. InSpace device (InSpace; OrthoSpace, Kfar Saba, Israel).

**Figure 13:**
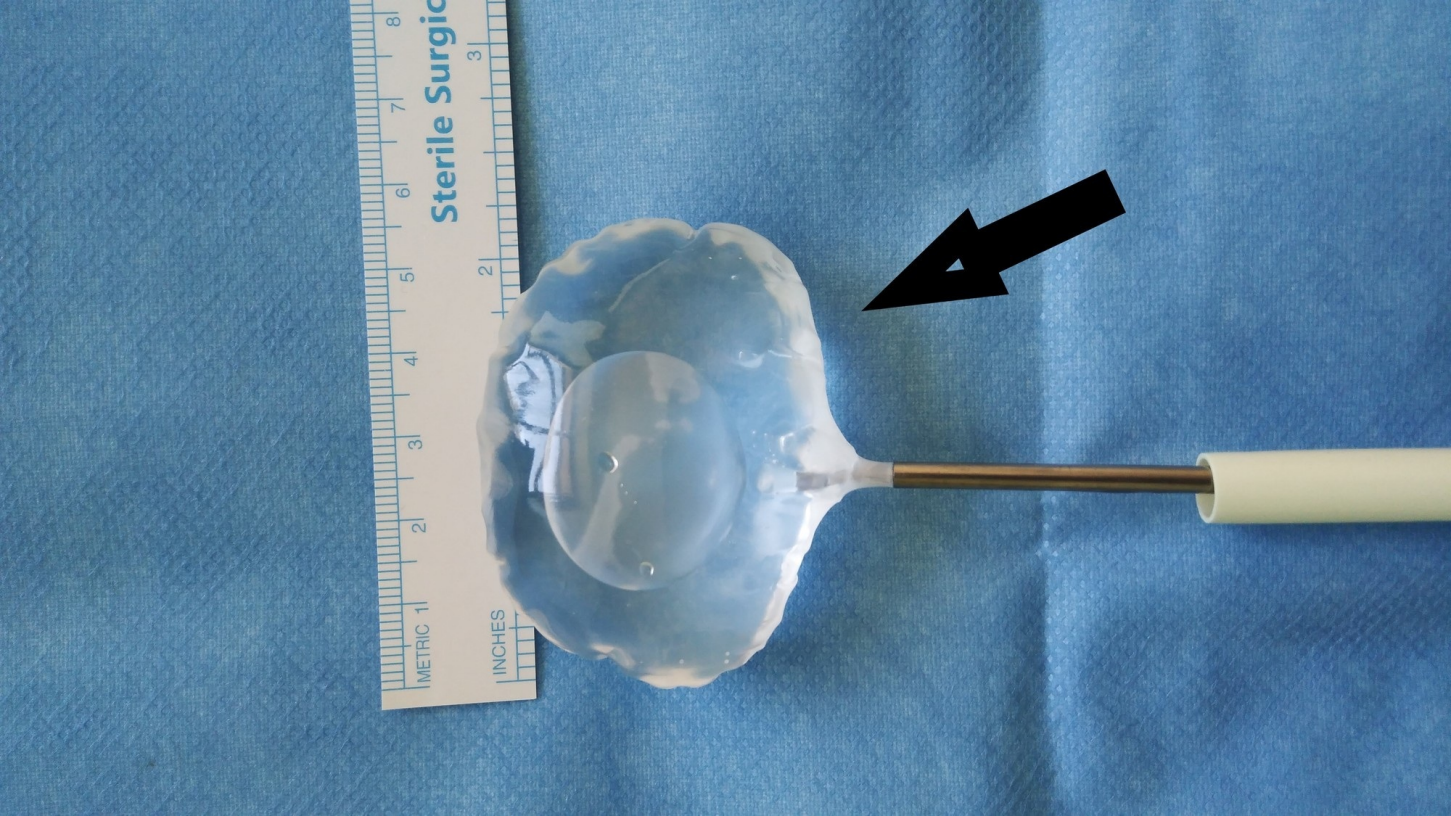
Mediolateral dimension About 5.5 cm mediolateral dimensions of the spacer balloon - in-vitro (medium-sized balloon). InSpace device (InSpace; OrthoSpace, Kfar Saba, Israel).

**Figure 14:**
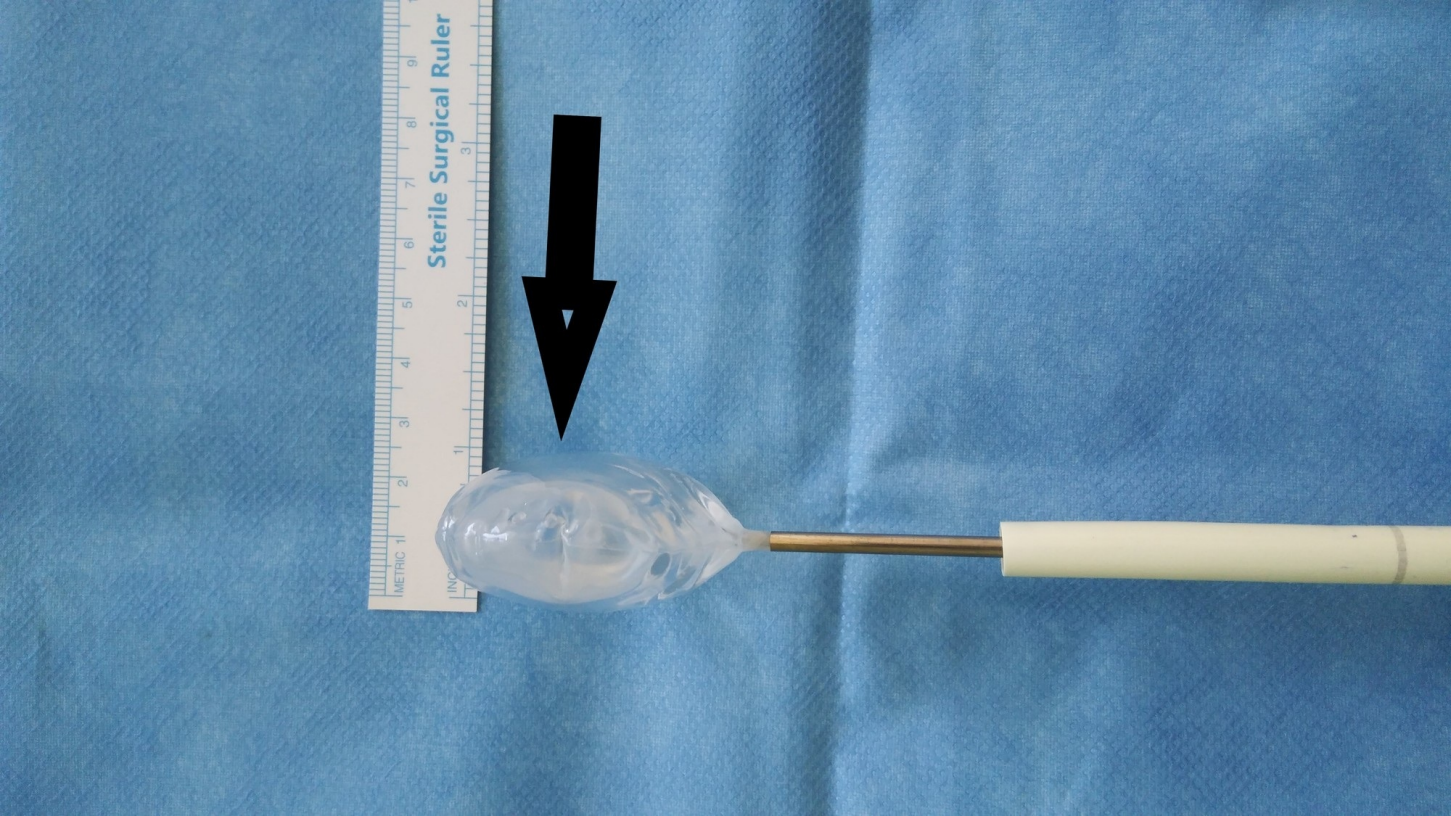
Thickness of medium-sized spacer balloon About 2 cm thickness of the medium-sized spacer balloon. InSpace device (InSpace; OrthoSpace, Kfar Saba, Israel).

**Figure 15:**
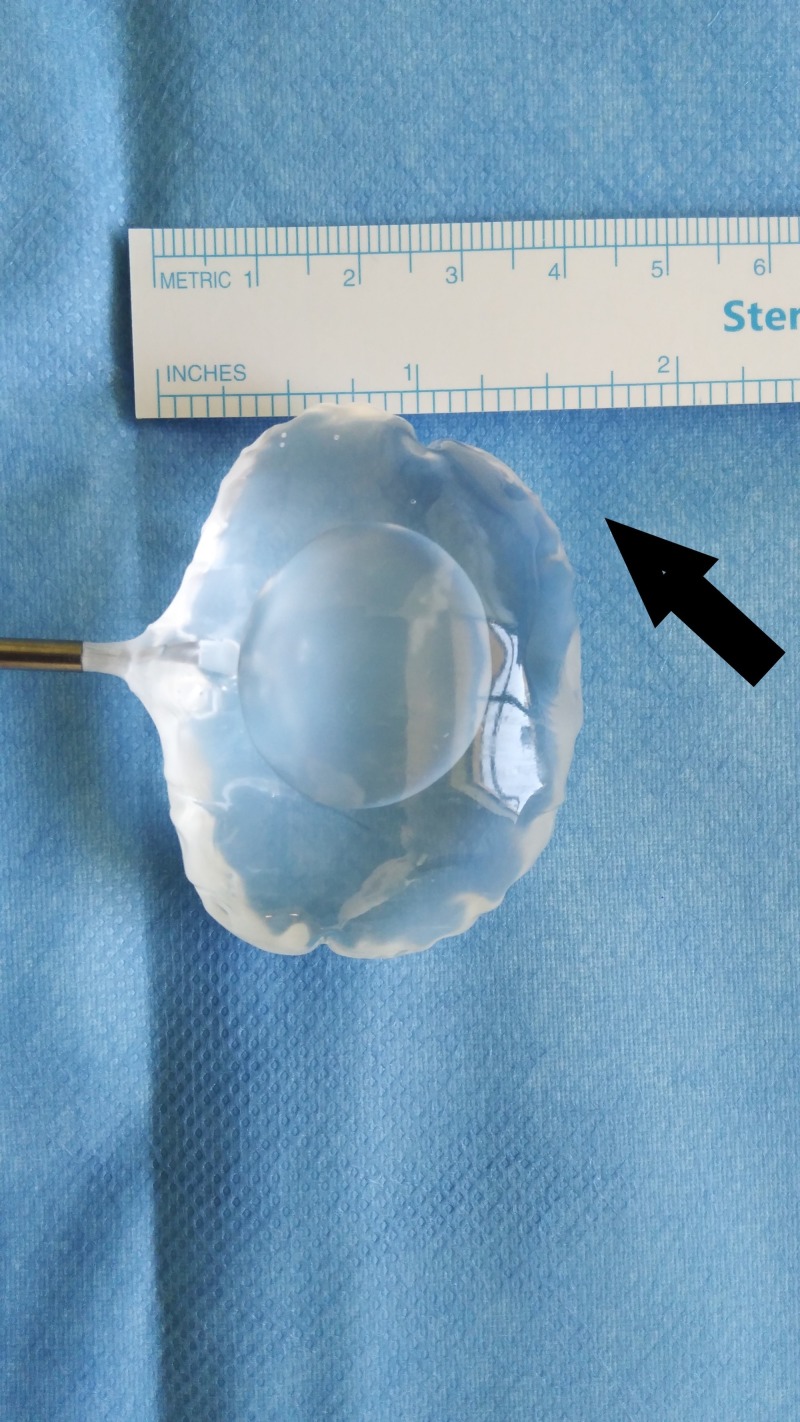
Anteroposterior dimensions About 4 cm anteroposterior dimension. InSpace device (InSpace; OrthoSpace, Kfar Saba, Israel).

Finally, the patient’s shoulder is moved around to determine proper function. The whole procedure, including standard arthroscopy and debridement, takes 15 to 20 minutes. The patient’s rehabilitation time is relatively short. The following in-vitro pictures show the spacer device, its components, and the unfolding of the balloon spacer (Figures 8-15 ).

## Discussion

Rotator cuff tears are quite debilitating. They cause severe pain and functional disability. Some massive tears in the rotator cuff, tears with poor quality muscle or tendon, and some massive chronic tears are very difficult to repair or if repaired, do not give the desired results. These refractory cases are the ones that are given the choice of either physiotherapy and analgesia or shoulder arthroplasty (Hemiarthroplasty, reverse shoulder arthroplasty, or cuff tear arthropathy prosthesis arthroplasty ) [3]. The first option of physiotherapy and analgesia works for a few patients and a majority of the patients have pain and limitation in function. On the other hand, shoulder arthroplasty is a major undertaking with its risks, complications, and huge and cautious demand for post-operative rehabilitation.

The spacer device gives a functional and practical option for the above-mentioned group of patients. It is inserted between the humeral head and the acromion via an arthroscope. It has shown to give good pain relief and improves shoulder function. The spacer device is biodegradable and degrades in six to 12 months [4].

## Conclusions

The spacer balloon (InSpace) device is a fairly simple, quick, and easy-to-learn procedure, which gives the patient with a rotator cuff injury good pain relief and improved shoulder movement with a generally short rehabilitation time.
